# Dual-Region Encryption Model Based on a 3D-MNFC Chaotic System and Logistic Map

**DOI:** 10.3390/e28020132

**Published:** 2026-01-23

**Authors:** Jingyan Li, Yan Niu, Dan Yu, Yiling Wang, Jiaqi Huang, Mingliang Dou

**Affiliations:** College of Computer Science and Technology (College of Data Science), Taiyuan University of Technology, Taiyuan 030024, China; 2023002047@link.tyut.edu.cn (J.L.); niuyan@tyut.edu.cn (Y.N.); yudan@tyut.edu.cn (D.Y.); 2023002052@link.tyut.edu.cn (Y.W.); 2023002032@link.tyut.edu.cn (J.H.)

**Keywords:** dual-region encryption, 3D-MNFC, MTCNN facial detection, CNN key generation, DNA encoding, dynamical analysis, security analysis

## Abstract

Facial information carries key personal privacy, and it is crucial to ensure its security through encryption. Traditional encryption for portrait images typically processes the entire image, despite the fact that most regions lack sensitive facial information. This approach is notably inefficient and imposes unnecessary computational burdens. To address this inefficiency while maintaining security, we propose a novel dual-region encryption model for portrait images. Firstly, a Multi-task Cascaded Convolutional Network (MTCNN) was adopted to efficiently segment facial images into two regions: facial and non-facial. Subsequently, given the high sensitivity of facial regions, a robust encryption scheme was designed by integrating a CNN-based key generator, the proposed three-dimensional Multi-module Nonlinear Feedback-coupled Chaotic System (3D-MNFC), DNA encoding, and bit reversal. The 3D-MNFC incorporating time-varying parameters, nonlinear terms and state feedback terms and coupling mechanisms has been proven to exhibit excellent chaotic performance. As for non-facial regions, the Logistic map combined with XOR operations is used to balance efficiency and basic security. Finally, the encrypted image is obtained by restoring the two ciphertext images to their original positions. Comprehensive security analyses confirm the exceptional performance of the regional model: large key space ($2^{536}$) and near-ideal information entropy (7.9995), NPCR and UACI values of 99.6055% and 33.4599%. It is worth noting that the model has been verified to improve efficiency by at least 37.82%.

## 1. Introduction

In the digital age, protecting privacy and security has become far more challenging, and image encryption has emerged as a critical puzzle. As key information carriers, images contain sensitive contents from personal private data to national-security-related information. Yet images can be leaked easily during transmission or storage and can be attacked or misused maliciously. Image encryption technology secures the confidentiality of sensitive information with strong protection: it works by breaking the inherent statistical patterns of images and boosting resistance to unauthorized decryption. This makes it indispensable for protecting individual rights and societal security. The field also faces unique challenges, primarily stemming from the large data volume and redundancy of image files.

Classic encryption algorithms like the Data Encryption Standard (DES), Advanced Encryption Standard (AES), RSA [1], and Diffie–Hellman (DH) [2] could hardly handle the complexity of image data. Chaotic systems present a promising alternative due to their sensitivity to initial values [3,4,5,6,7,8,9]. For instance, a study proposed a chaotic system (TLCMCML) and applied it to a multi-image medical encryption algorithm [10]. A novel fractional-order delayed chaotic system was proposed and applied to image encryption [11]. Zhang et al., introduced a method for image encryption that utilizes an enhanced Lorenz chaotic system in conjunction with Galois field [12]. A facial image privacy protection approach based on 2D-DMHM and DNA cryptography demonstrated superior security and efficiency [13]. Furthermore, significant progress has been achieved in the application of chaotic systems in image encryption: based on memristor-based multi-scroll Hopfield neural networks, dynamic analysis and FPGA implementation have enhanced the hardware deployment capability of chaotic systems [14]; the fractional-order memristive Hopfield neural network has introduced hidden chaotic double-wing attractors, strengthening the complexity of the system [15]; the state-dependent variable fractional-order hyperchaotic system, by coupling quadratic maps, has provided a new scheme for high-performance image protection [16]; the two-dimensional coupled enhanced cubic hyperchaotic map, combined with exponential parameters, has achieved hierarchical saliency-aware multi-image encryption [17]. These studies have contributed numerous methods to the application of chaotic systems in encryption, yet the issue of fixed parameters remains unresolved, resulting in insufficient ability to resist analytical attacks. The proposed 3D-MNFC is introduced with time-varying parameters to solve this problem and added coupling mechanism to enhance the randomness.

Images that only use chaotic systems for encryption usually show weak robustness when subjected to interference attacks such as cropping. To solve this defect, the combination of chaotic systems with other disciplines has become a popular trend. A hybrid approach for medical image encryption that merges DNA-based encoding technology with elliptic curve cryptography has been described [18]. Deep learning models have also been applied to encryption, and Kirtee Panwar and his partners summarized the trends of image encryption based on it [19]. Based on the above analysis, this kind of combination seems to exhibit better performance [20,21,22,23,24,25,26,27]. Therefore, a complex encryption method integrating chaotic systems and other operations is required to protect facial regions, as they are highly sensitive. The constructed model, integrating a CNN-based generator, the proposed 3D-MNFC, DNA encoding and bit reversion, significantly ensures the security of facial regions. Cryptanalysis research also provides important references for the design of encryption schemes: For encryption systems based on binary bit-plane extraction and multi-chaotic maps, researchers have found that the diffusion and permutation parts can be cracked separately by the divide-and-conquer strategy [28]; a color image encryption algorithm based on the fractional-order hyperchaotic system (CIEA-FOHS) can be broken by chosen-plaintext attack methods [29]; encryption schemes based on Feistel networks and dynamic DNA encoding have been shown to have several flaws in the design of keys, chaotic sequences, Hill encryption, and other aspects [30]. To address these attack types, this study proposes a dual-region encryption model that integrates a CNN-based generator, the 3D-MNFC, DNA encoding, and bit reversion, thereby significantly ensuring the security of facial regions. Through NPCR/UACI analysis, information entropy analysis, and resilience tests against cropping/noise attacks, the model is verified to possess sufficient practicality and security.

In addition, there is still an efficiency problem. Striking a balance between computational complexity and security performance is essential. Segmenting the image and encrypting each region as needed is one solution. It is more time-intensive and computationally demanding to encrypt the whole image compared to encrypting regionally. However, most existing studies focus on uniform encryption of the entire image [31,32,33], and multi-region encryption algorithms based on face detection are rarely mentioned. As a result, an encryption model is required to align various performance metrics closely with their optimal levels and minimize computational time.

**Figure 1:**
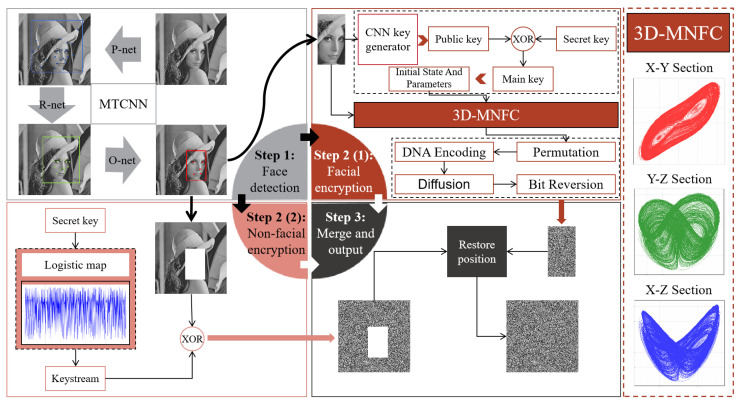
Workflow of the dual-region encryption model for portrait images.

To address the above needs, this paper proposes a dual-region encryption model. The encryption model detects the facial areas in the image through a Multi-task Cascaded Convolutional Network (MTCNN). For the facial region, 3D-MNFC is proposed in this study. Built upon the Lorenz and Chen systems, 3D-MNFC incorporates time-varying parameters, nonlinear terms and state feedback terms. Through analyses of various metrics, it has been verified to possess complex dynamic characteristics. After generating the public key via the CNN key generator, the encryption process for the facial region takes 3D-MNFC as its core and combines it with multi-level encryption operations to achieve secure encryption. The encryption for non-facial regions is relatively simple: a key stream is generated by the Logistic map and encryption is carried out through XOR operations. The workflow of this encryption model is illustrated in Figure 1. Analysis of this encryption model shows that it has good robustness and reduces encryption time.

The contributions of this model can be summarized as follows:

(1) A novel three-dimensional Multi-module Nonlinear Feedback-coupled Chaotic System (3D-MNFC) is proposed, which has been verified to exhibit complex chaotic characteristics through a series of rigorous tests.
(2) A new method which integrates a CNN-based key generator, the proposed 3D-MNFC, DNA encoding, and bit reversion is proposed. It provides robust security for facial regions.
(3) We propose a composite dual-region encryption model. After undergoing a comprehensive suite of analyses and comparisons, this model demonstrates a balance between computational efficiency and security performance.

Finally, the remaining sections of this paper are described as follows: Section 2 introduces some fundamental theories. Section 3 proposes and analyzes 3D-MNFC. Section 4 elaborates the dual-region encryption model. Section 5 conducts a security analysis of the model. Section 6 summarizes the main contributions of this work and discusses potential directions for future research.

## 2. Fundamental Theories

This section introduces the fundamental theories relevant to this research, including two classical chaotic systems and the application of DNA encoding in encryption, providing theoretical support for the subsequent design of 3D-MNFC and the construction of the encryption model.

### 2.1. Chaotic System

Image encryption is a core part of information security. It is applied to break down the inherent pixel relationships and statistical patterns in images through advanced scrambling and diffusion. Nowadays, most image encryption algorithms are based on chaotic systems. One standout example is the use of a quadratic polynomial hyperchaotic map [34]. This approach has acquired impressive results, specifically achieving extremely high levels of information entropy. Furthermore, Nan-Run Zhou and his colleagues developed an encryption scheme based on bit-level expansion [35], which has proven resilient across various test conditions. A lightweight image encryption method [36] tailored for IoT networks built on multiple chaos systems was also introduced. This method holds up well against pixel intensity modification attacks. Although these methods yield satisfactory results in certain areas, fixed parameter settings impose an inherent restriction on their flexibility. The field still needs an encryption algorithm that can smoothly balance multiple key indicators. It can ensure comprehensive security while avoiding any compromise on efficiency.

The Lorenz and Chen systems are classical nonlinear dynamical models in chaos theory. They were known for their complex chaotic dynamics. For this reason, the encryption model we propose in this paper uses 3D-MNFC based on these two frameworks. The Lorenz system (1) is defined as follows:

(1) $$\left\{\begin{array}{c} \frac{dx}{dt}=\sigma \left(y-x\right)\\ \frac{dy}{dt}=x\left(\rho -z\right)-y\\ \frac{dz}{dt}=xy-\beta , \end{array}\right.$$

where *x* is convection intensity; *y* is horizontal temperature gradient; *z* is vertical temperature gradient; and σ, ρ, β are system parameters. When σ = 10, ρ = 28, and β = 8/3, the system enters a chaotic state.

The Chen system (2) is defined as follows:

(2) $$\left\{\begin{array}{c} \frac{dx}{dt}=a\left(y-x\right)\\ \frac{dy}{dt}=\left(c-a\right)x-xz+cy\\ \frac{dz}{dt}=xy-bz, \end{array}\right.$$

where *a*, *b*, *c* are system parameters. When *a* = 35, *b* = 3, and *c* = 28, the system exhibits typical chaotic behavior.

### 2.2. DNA Encoding

In the field of biology, deoxyribonucleic acid (DNA) molecules exhibit a double-helix structure, composed of four nucleotide bases: adenine (A), thymine (T), guanine (G), and cytosine (C). The vast diversity of DNA base sequences serves as the molecular basis for biological diversity, with well-established base-pairing rules—A forms complementary pairs with T, and C forms complementary pairs with G.

For encryption applications, these four bases can be mapped to 2-bit binary values (e.g., A to “00”, C to “01”, G to “10”, and T to “11”). This study adopts the encoding rules detailed in Table 1, where each rule corresponds to a unique base permutation strategy.

There is the workflow of DNA-encoded transformation. Each pixel value ranging from 0 to 255 is converted into an 8-bit binary string. That string is divided into four smaller 2-bit substrings. Each of these 2-bit chunks gets mapped to a corresponding DNA base, using whatever encoding rule was picked for the process. A 4-base DNA sequence that acts as a stand-in for the original pixel is generated. The next move is to scramble them. The key used here comes from the chaotic system that is initialized with the public and private keys the CNN generates. This scrambling step matters a lot because it breaks up the spatial correlation in the pixel data, and that is a big part of hiding the original structure of images so it cannot be easily decoded. The last step is reversing the mapping on those scrambled DNA sequences to turn them back into 8-bit binary strings. By converting those binary strings to decimal values, they are exactly the encrypted pixels.

Decryption is the reverse of the above process. Taking the encrypted pixels and converting them back to DNA sequences, we use the same chaotic key to unscramble those sequences to retrieve the original DNA sequences. Then, the mapping is reversed again to be turned into binary strings, and those strings are converted to decimal pixel values. Crucially, we must use the exact same encoding rule used for encryption.

## 3. The Proposed 3D-MNFC

This section introduces the composition of the proposed 3D-MNFC and shows the tests related to it. The results declare that it has good chaos and sufficient randomness.

### 3.1. The Composition of 3D-MNFC

A three-dimensional Multi-module Nonlinear Feedback-coupled Chaotic System (3D-MNFC) is constructed:

(3) $$\left\{\begin{array}{c} x=\left(1-\alpha \right)L_{x}+\beta H_{x}\\ y=(L_{y}+\alpha C_{y})/2\\ z=\alpha C_{z}, \end{array}\right.$$

where *x*, *y*, *z* are state parameters; α is a weighted coefficient; and β serves as a variable control parameter. *L* and *C* refer to the modified Lorenz system and Chen system. Their specific formulations are detailed as follows:

(4) $$\left\{\begin{array}{c} L_{x}=a_{t}\left(y-x\right)+\beta F_{y}\\ L_{y}=c_{t}x-y-xz\\ L_{z}=xy-bz, \end{array}\right.$$

(5) $$\left\{\begin{array}{c} C_{x}=a_{t}\left(y-x\right)\\ C_{y}=\left(c_{t}-a_{t}\right)x-xz+c_{t}y\\ C_{z}=xy-dz+\beta G_{z}, \end{array}\right.$$

where $a_{t}$ and $c_{t}$ are time-varying parameters designed to address the periodic limitation of fixed-parameter chaotic systems. Specifically, we introduce periodic perturbation terms (sine/cosine functions) to modulate the base parameters of Lorenz and Chen subsystems: the low-frequency terms (0.05*t*, 0.02*t*, etc.) simulate the natural nonlinear fluctuations (e.g., fluid vibration), while the weight coefficients (0.3, 0.2, etc.) control the perturbation amplitude to prevent the system from falling into periodic orbits. Physically, $a_{t}$ corresponds to the convection intensity of the Lorenz subsystem, and $c_{t}$ corresponds to the horizontal temperature gradient of the Chen subsystem. The time-varying nature of $a_{t}$ and $c_{t}$ breaks the thermal equilibrium of the system, expanding the phase space trajectory and enhancing the unpredictability of chaotic behavior. $H_{x}$ and $F_{y}$ represent the nonlinear cross terms and $G_{z}$ is the state feedback term. They are designed to generate high-complexity chaotic sequences for image encryption, with the core goal of enhancing randomness and avoiding periodicity. The specific formulations are detailed as follows:

(6) $$\left\{\begin{array}{c} t_{1}=sin\left(0.05\times t\right)\times 0.3+cos\left(0.02\times t\right)\times 0.2+1.0,\\ t_{2}=cos\left(0.07\times t\right)\times 0.4+sin\left(0.03\times t\right)\times 0.1+1.0,\\ a_{t}=at_{1},\\ c_{t}=ct_{2}, \end{array}\right.$$

(7) $$\left\{\begin{array}{c} H_{x}=0.2\cdot x\cdot \left(1-\frac{x^{2}}{1000}\right),\\ F_{y}=e\times \frac{y^{2}\left(z-x\right)}{0.3+y^{2}}+\beta \left[sin\left(y\right)cos\left(x+y\right)+sin\frac{y}{3}cos\frac{x}{4}\right],\\ G_{z}=kztanhytanhx+0.1z\cdot e^{-0.01(x^{2}+y^{2})}. \end{array}\right.$$

In Equation (7), k is a state feedback intensity adjustment parameter, whose core function is to dynamically adjust the weight of the state feedback term, avoiding system oscillation caused by excessive feedback or insufficient randomness caused by weak feedback. The term kztanhytanhx performs nonlinear compression on state variables x and y through hyperbolic tangent functions tanhy and tanhx (output range −1∼1), then couples with state variable z to enhance the nonlinear correlation between system states. Combined with the subsequent exponential decay term $0.1z\cdot e^{-0.01(x^{2}+y^{2})}$, it jointly suppresses the system from falling into periodic orbits, improves the random characteristics of chaotic sequences.

As a result, a high-performance 3D-MNFC is established. This chaotic system significantly mitigates the limitation of vulnerability to brute-force attacks, which arises from the inflexible parameter of traditional chaotic systems.

### 3.2. Lyapunov Exponent and Trajectory

**Figure 2:**
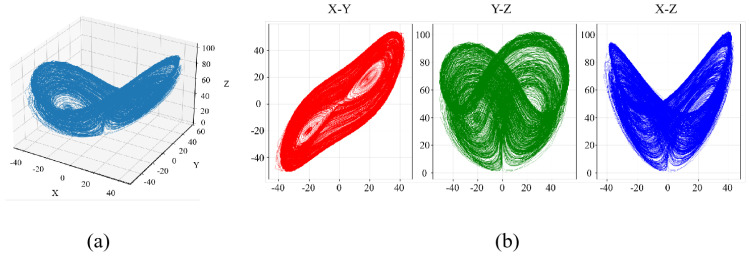
(**a**) The 3D trajectory diagram of 3D-MNFC; (**b**) the phase distributions of the attractor.

**Figure 3:**
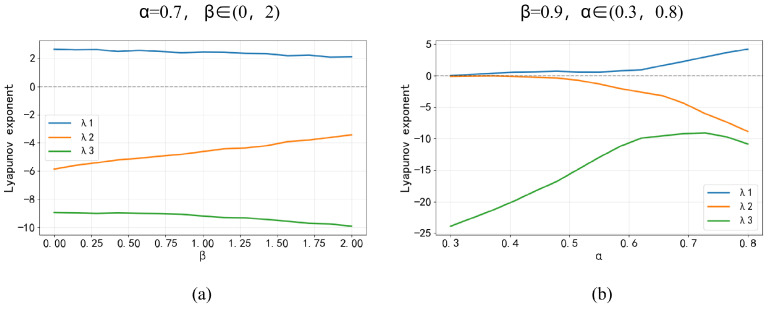
Lyapunov exponent diagrams with (**a**) α = 0.7, β∈(0,2) and (**b**) β = 0.9, α∈(0.3,0.8).

The phase distribution of a chaotic system can intuitively reflect the complexity of the system’s chaotic behavior. The Lyapunov exponent, a core characteristic of chaotic systems, is used to quantify the degree of divergence or convergence of system trajectories. A positive Lyapunov exponent value is generally recognized as a key criterion for determining the presence of chaotic behavior in a system. Figure 2 presents the 3D trajectory diagram of 3D-MNFC proposed in this study, along with the phase distributions of the attractor across different planes. Figure 3 shows the Lyapunov exponent diagram of the proposed 3D-MNFC.

### 3.3. Bifurcation Diagram

**Figure 4:**
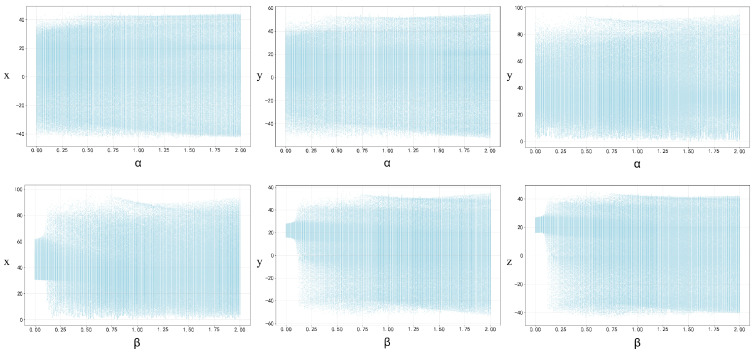
Bifurcation diagrams.

Different parameters exert considerable impacts on chaotic systems, and bifurcation diagrams are capable of illustrating the dynamic characteristics exhibited by such parameter variations. When the initial states $x_{0}=y_{0}=z_{0}=0.1$, the control parameters α and β fall within the interval (0, 2), and the ergodicity of the proposed 3D-MNFC is demonstrated in Figure 4.

### 3.4. Permutation Entropy

Permutation entropy (PE), proposed by Bandt and Pompe [37], is a quantitative index for measuring the complexity of nonlinear time series. It converts continuous time series into discrete permutation patterns and evaluates the complexity of the series based on the uniformity of pattern distribution. The more uniform the pattern distribution, the stronger the randomness of the series, and consequently, the higher the entropy value. The calculation formula for permutation entropy is expressed as (8)

(8) $$PE=-\sum_{i=1}^{m}P_{i}lnP_{i}.$$

Normalizing it to the interval [0, 1] yields (9)

(9) $$PE=\frac{PE}{ln(m!)}.$$

**Figure 5:**
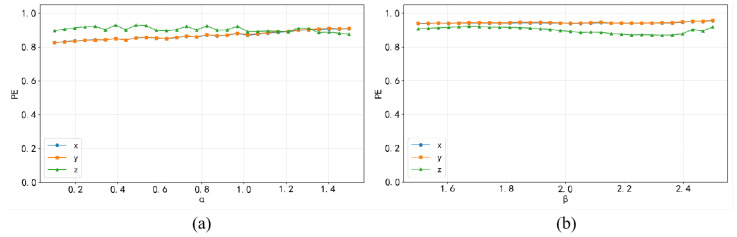
PE with (**a**) β = 0.9, α∈(0,1.5) and (**b**) α = 0.7, β∈(0,2.5).

Figure 5 presents the permutation entropy analysis of 3D-MNFC, and the results demonstrate that the sequences generated by it exhibit higher unpredictability.

### 3.5. 0-1 Test

**Figure 6:**
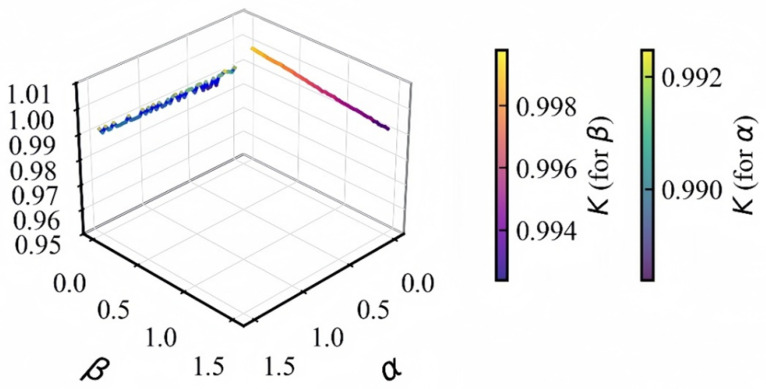
K values of 0-1 test.

The 0-1 test, a chaos identification method developed by Gottwald and Melbourne, is used to determine whether chaotic behavior exists in a deterministic time series. To implement this test for a chaotic sequence *f*, the following formula is applied (10):

(10) $$\left\{\begin{array}{c} p\left(n\right)=\sum_{i=1}^{n}\phi \left(i\right)cos\left(ci\right),\\ q\left(n\right)=\sum_{i=1}^{n}\phi \left(i\right)sin\left(ci\right),\\ M\left(n\right)=\lim_{n\to \infty }\frac{1}{N}\sum_{i=1}^{N}\left[{\left(p\left(n+i\right)-p\left(i\right)\right)}^{2}+{\left(q\left(n+i\right)-q\left(i\right)\right)}^{2}\right],\\ K=\frac{logM(n)}{logn}, \end{array}\right.$$

where *N* is one-tenth of the sequence length. If the test statistic *K* converges to 0, it indicates that the system exhibits regular motion; if *K* converges to 1, this confirms the presence of chaotic behavior in the system. Figure 6 presents the 0-1 test results for the proposed 3D-MNFC. The range of the *K* value indicates that 3D-MNFC exhibits an extremely high degree of chaotic behavior.

The parameter α was set within the range of 0.10 to 1.50, and its corresponding K values were distributed between 0.988382 and 0.992469, with an average value of 0.9908. There was no obvious monotonic trend in the overall K value variation, but all values remained close to 1, with a local minimum observed only at α = 0.30. For parameter β, which was also tested in the range of 0.10 to 1.50, its K values exhibited a clear monotonic decreasing trend, gradually declining from 0.999777 to 0.992389, with an average value of 0.9958. All the obtained K values were significantly higher than the chaos judgment threshold of 0.95, which verifies that the system maintains robust chaotic characteristics within the tested parameter ranges of α and β. The results indicate that 3D-MNFC exhibits an extremely high degree of chaotic behavior.

### 3.6. NIST SP 800-22 Test

NIST SP 800-22 [38] is a standard issued by the National Institute of Standards and Technology (NIST) for the evaluation of random number and pseudorandom number generators. This test comprises 15 individual test items, and each item yields a *p*-value. Only when the *p*-value exceeds 0.01 can the randomness of the random number sequence be deemed sufficient for application requirements. The test results presented in Table 2 demonstrate that the 3D-MNFC has passed the NIST SP 800-22 test, confirming its applicability for image encryption.

## 4. The Proposed Encryption Model

In this section, we introduce the MTCNN model utilized. Then we delve into the encryption method for facial regions and non-facial regions. After integrating the above processes to construct the overall encryption model, we provide the decryption algorithm.

### 4.1. Face Detection Based on MTCNN

**Figure 7:**
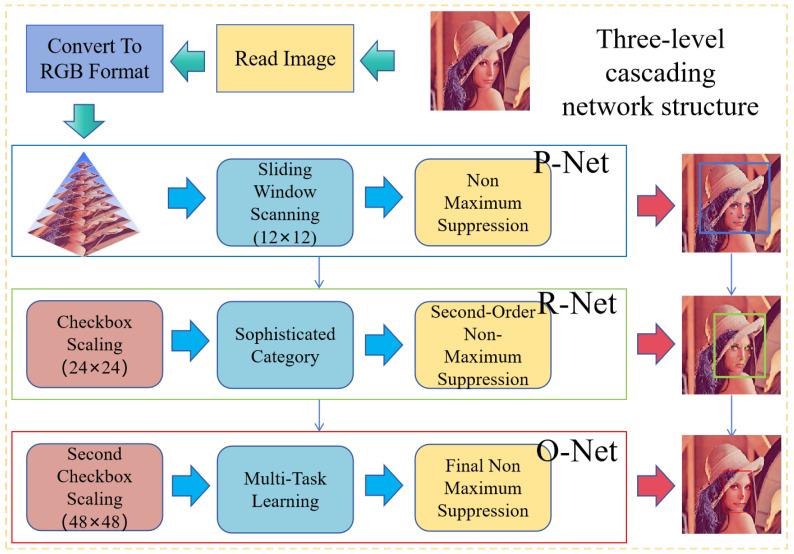
The three-stage cascaded network structure of MTCNN.

Face detection has long been a cornerstone of computer vision research. It involves identifying and pinpointing the location of human faces accurately for input images. In recent years, there have been rapid advances in this technology, with many innovative methods emerging [39,40,41]. As a Multi-task Cascaded Convolutional Network tailored specifically for face detection and facial key point localization, MTCNN has a three-stage cascaded network structure [42]. Its cascaded architecture is shown in Figure 7. This architecture can extract key facial features layer by layer from low-resolution images, expanding the application scenarios of the encryption algorithm. In addition, MTCNN supports the customization of the $\min_{facesize}$ parameter, which can flexibly adapt to the minimum face size under different resolutions and thus meets the requirement for accurate localization of facial regions. Moreover, MTCNN can locate facial key points during the detection process instead of only returning face bounding boxes, which enables fine-grained segmentation of facial regions. Compared with lightweight and efficient models or accurate models with more complex architectures, MTCNN to strike a balance between high accuracy and fast performance in complex scenarios. Zhang et al. verified that it can obtain the position of face more accurately [43]. And it has a higher operational efficiency [42], being able to achieve real-time recognition [44]. Hence, the face detection based on MTCNN was applied to the proposed encryption model, aiming at balancing the accuracy and efficiency.

The Proposal Network (P-Net) is a streamlined convolutional model that spots potential facial regions and pulls initial facial key points. It resizes the input image across multiple scales, which builds out an image pyramid. A 12 × 12 sliding window scans each layer of the pyramid. Each window goes through binary classification and bounding box regression. The likelihood of a face being present and the necessary offsets can be delivered. Non-Maximum Suppression (NMS) then refines these outputs: it filters out low-confidence candidate boxes to lock in approximate facial region locations and preliminary key point data.

The Refine Network (R-Net) is key to sharpen face detection outcomes. It starts with the candidate regions identified by P-Net, maps them back to the original image, then crops and resizes each one to a fixed 24 × 24 input dimension. It runs a second round of classification and bounding box regression by using a more complex network structure. After that, NMS and threshold checks are applied to filter the results, leaving only refined face boxes. The result is fewer false detections, along with a noticeable boost in overall accuracy.

The Output Network (O-Net) is the final stage, undertaking the tasks of high-precision face bounding box localization and facial key point detection. It maps the candidate boxes output by the R-Net back to the original image and processes them into 48 × 48 inputs. With the help of a deeper network structure, it simultaneously accomplishes face classification, bounding box regression, and the localization of five key points (both eyes, the tip of the nose, and the corners of the mouth). After non-maximum suppression, it outputs the precise localization results that can be used for subsequent tasks.

**Figure 8:**
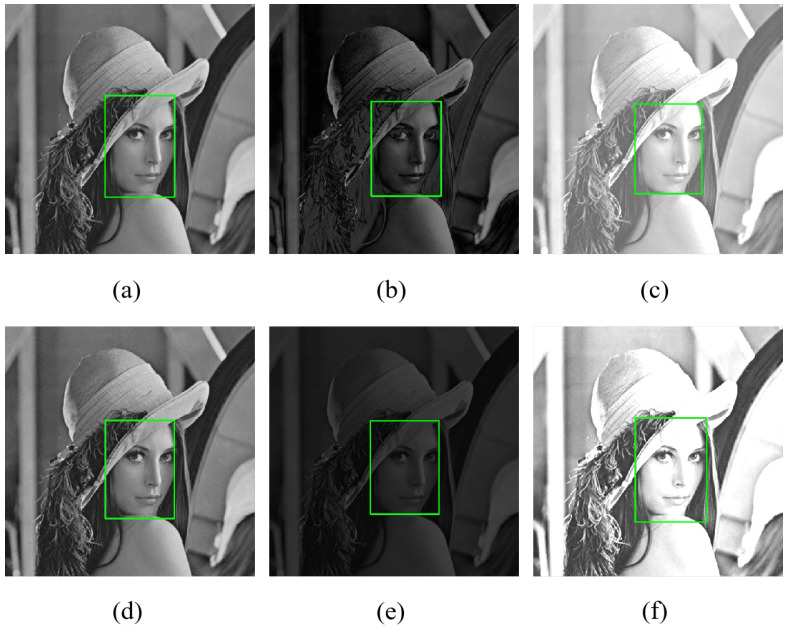
Detection results under (**a**) original image; (**b**) low brightness; (**c**) high brightness; (**d**) Gaussian noise; (**e**) low contrast; (**f**) high contrast.

To defend against some extreme attacks, we conduct face detection on the test images under the conditions of original image, low brightness, high brightness, low contrast, high contrast, and Gaussian noise, respectively. Figure 8 shows the detection results of the Lena image under the above conditions. Table 3 shows the number of faces detected by MTCNN in images under different environments, which demonstrates that it can accurately detect faces in most cases. Therefore, it is sufficient for face detection before encryption.

### 4.2. Encryption for the Facial Region

#### 4.2.1. CNN Key Generation

Erkan, U. integrated deep learning into key generation [45], so we used a deep Convolutional Neural Network (CNN) key generation model. The core idea here is to pull out high-level semantic features from the input image by the CNN. These features then go through transformation and binarization to create a binary public key. It not only ensures the key has a strong link to the image but also boosts its randomness and uniqueness.

SHA-256 generates keys solely based on the hash values of images, and its sensitivity to minor image variations is lower than that of CNN-based feature extraction. Therefore, we only use it as a seed rather than directly generating keys with it. Networks such as InceptionNet and ResNet contain numerous complex structures, which may lead to unstable key generation logic. Given that this study aims to improve encryption efficiency, lightweight networks appear to be the optimal choice. However, lightweight networks like MobileNet have relatively low feature dimensions, resulting in weak correlation between the generated keys and image content, which is prone to key collision. Clearly, the demand for strong key–content correlation takes precedence over the pursuit of speed. As a result, we chose VGG16 as our base feature extraction network to accomplish it. The fact that it is pre-trained on large-scale datasets gives it a strong track record of capturing multi-scale image features. Maps with multiple dimensions that come out of its convolutional layers do a solid job of capturing the input image’s intrinsic attributes. This is a major advantage, because it lets us pick up on subtle differences between different images.

**Figure 9:**
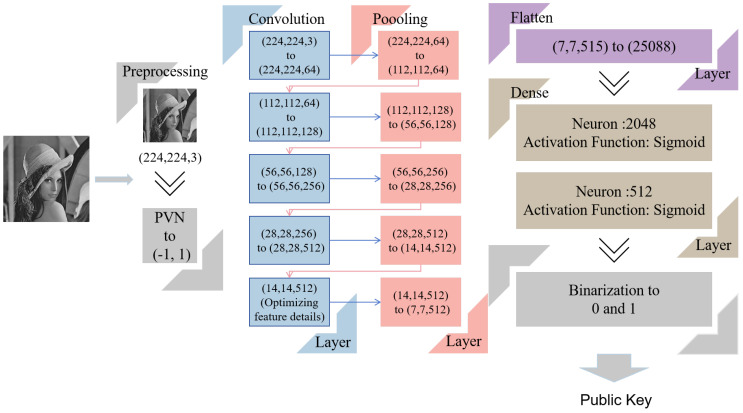
CNN public key generation.

To tweak VGG16 for public key generation, we stripped out the original classification layers at the top of the network. Then a custom feature transformation module was added: a Flatten layer was used to turn the convolutional feature maps into a one-dimensional vector. Next, we fed that vector into two back-to-back dense layers. These handle dimensionality reduction and non-linear transformation. The first dense layer spits out a 2048-dimensional feature vector, and the second one compresses that down to 512 dimensions. Both of them used sigmoid activation. The 512-dimensional vector is the base for generating the public key. Figure 9 lays out the full workflow in detail.

The extracted features can ensure not only a strong correlation between the key and the image but also the uniqueness of the key. The network structure of VGG16, pre-trained on large-scale datasets, enables its shallow convolutional layers to capture low-level visual features such as image edges and textures, while the deep convolutional layers can abstract high-level semantic features including facial contours. These features are directly determined by image content without redundant information irrelevant to the image, and the unique facial attributes of portrait images will form exclusive feature vectors in the deep feature maps, laying a foundation for the strong correlation between the key and the image. Meanwhile, the features extracted by VGG16 reach an initial dimension of 25088 after transformation via the Flatten layer, which is then compressed to 512 dimensions through two fully connected layers. The collision probability of feature vectors between any two different images in the high-dimensional space is close to zero. Additionally, the fully connected layers adopt the Sigmoid activation function for nonlinear transformation, which further amplifies the subtle differences between features of different images. Even for portrait images with similar content, differentiated feature vectors can be generated, ensuring the uniqueness of the key. Furthermore, the features extracted by VGG16 reflect the comprehensive information of the global semantics and local details of the image, rather than a simple mapping of individual pixels or local regions. Attackers can hardly forge feature vectors consistent with the original image by tampering with local pixels of the image. The content relevance of the feature vectors can be directly transmitted to the final key, endowing the key with an “image-binding characteristic”—only the features of the corresponding image can generate valid keys, thus guaranteeing the directionality and security of encryption.

The above introduces how the CNN-based key generator works, and the following information is on applying it to actual encryption. When encryption starts, it produces a content-dependent public key—with the SHA-256 hash value serving as the seed. And a random private key is concurrently generated to generate a main key through XOR. Specifically, the facial region employs a dual-key architecture composed of a public key and a private key, which are combined to form the main key. The main key is then subjected to matrix transformation to extract the initial values and control parameters for the chaotic system.

#### 4.2.2. Operations Based on 3D-MNFC

A sophisticated mechanism that integrates the 3D-MNFC, DNA encoding, and bit reversion is employed to encrypt facial regions. After obtaining the initial values and control parameters for the chaotic system, the encryption process proceeds in four steps:

Step 1. Permutation: The 3D-MNFC system extracts initial values ($x_{0}$ = $y_{0}$ = $z_{0}$ = 0.1) and control parameters (α = 0.7, β = 0.9) from the master key generated by CNN and iterates to generate a 3D chaotic sequence. After normalizing to the pixel coordinate range, a permutation index matrix is constructed to realize the random mapping of pixel positions through a row–column permutation algorithm. This step disrupts the spatial correlation of the original image.

Step 2. DNA encoding: One of the eight rules of DNA encoding is dynamically selected. Each 8-bit binary value of pixel is converted into a DNA sequence, and it is then remapped back to a pixel value.

Step 3. Diffusion: The chaotic sequence generated by 3D-MNFC is normalized (0–255) and rounded to generate a keystream, which performs bitwise XOR operation with the pixel values after DNA encoding. This ensures that minor changes in individual pixels are diffused across the entire facial region to eliminate localized statistical patterns.

Step 4. Bit Reversion: A bit reversal mask is constructed based on the parity of the chaotic sequence (e.g., reversing the first 4 bits when the sequence value is positive, and reversing the last 4 bits when negative). The bit reversal operation is performed on the binary string of the diffused pixels, and then a second XOR operation is performed with another set of chaotic keystreams. This step further enhances encryption strength by introducing an additional layer of complexity.

Relevant information about the encrypted image of the facial region is preserved, which will be utilized in the subsequent fusion of the two regions.

### 4.3. Encryption for the Non-Facial Region

Non-facial regions have relatively lower security requirements. Thus, we adopt an encryption scheme with fewer steps. It combines the Logistic chaotic map with XOR operation. The Logistic map is used to generate chaotic sequences by (11):

(11) $$x_{n+1}=\mu x_{n}\left(1-x_{n}\right),$$

where μ is the control parameter. The map exhibits complex chaotic behavior when 3.5699 < μ ≤ 4. This map significantly reduces computational work quite a bit. During encryption, this region adopts a single-key architecture, where a 3-byte private key is used to control the map’s initial values, parameters, and random seed. Once the chaotic sequence is generated, it does not work alone. Instead, it teams up with a random mask. These two steps work together to create the keystream. This keystream runs an XOR operation with the pixels in non-facial areas to accomplish encryption.

This method takes simple measures, but it can still resist common attacks. It markedly improves encryption efficiency. This advantage is more pronounced in images where non-facial regions account for a large part.

### 4.4. The Complete Image Encryption Model

We employ the techniques described earlier to elaborate on the process of the dual-region encryption model.

The model utilizes MTCNN to detect human faces in the image and obtain the bounding box coordinates of these facial regions. The image is segmented into facial and non-facial regions according to these coordinates. Subsequently, the two regions undergo independent encryption processes: For the facial region, a CNN-based key generator is first used to obtain a public key, which is then combined with a randomly generated private key to produce a main key. The 3D-MNFC system extracts initial values and control parameters based on the main key, iterates 500 times to generate a three-dimensional chaotic sequence, and then converts it into the encryption sequence after normalization and rounding. Thereafter, the high-security encryption is implemented using the approach proposed in Section 4.2, which integrates the CNN key generator, the 3D-MNFC, DNA encoding, and bit reversion. For the non-facial region, the Logistic map determines the initial values, control parameters, and random seeds by means of a 3-byte private key, and after iteratively generating a chaotic sequence, performs a modulo-256 addition operation with a random mask to produce the encryption sequence. In accordance with the method outlined in Section 4.3, rapid encryption is achieved through the Logistic map and XOR operation. Finally, the encrypted facial and non-facial regions are restored to their original positions in the image, resulting in a fully encrypted image.

### 4.5. Decryption Algorithm

**Figure 10:**
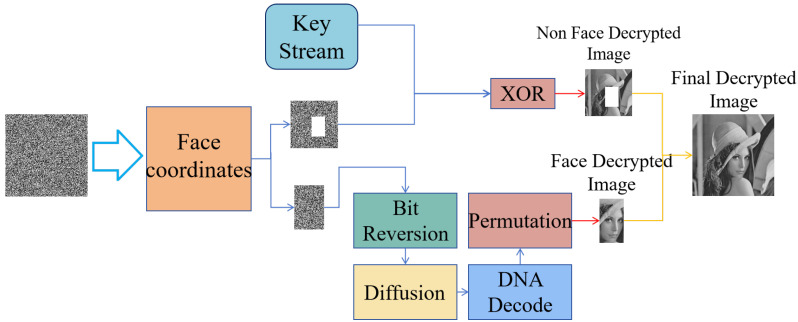
The process of decryption.

Based on the encryption model described above, the decryption algorithm is a completely reverse process, as shown in Figure 10.

For decrypting the non facial region, a keystream is generated using the encryption key. By performing an XOR operation between this keystream and the encrypted non facial image, the original non-facial image can be restored.

**Figure 11:**
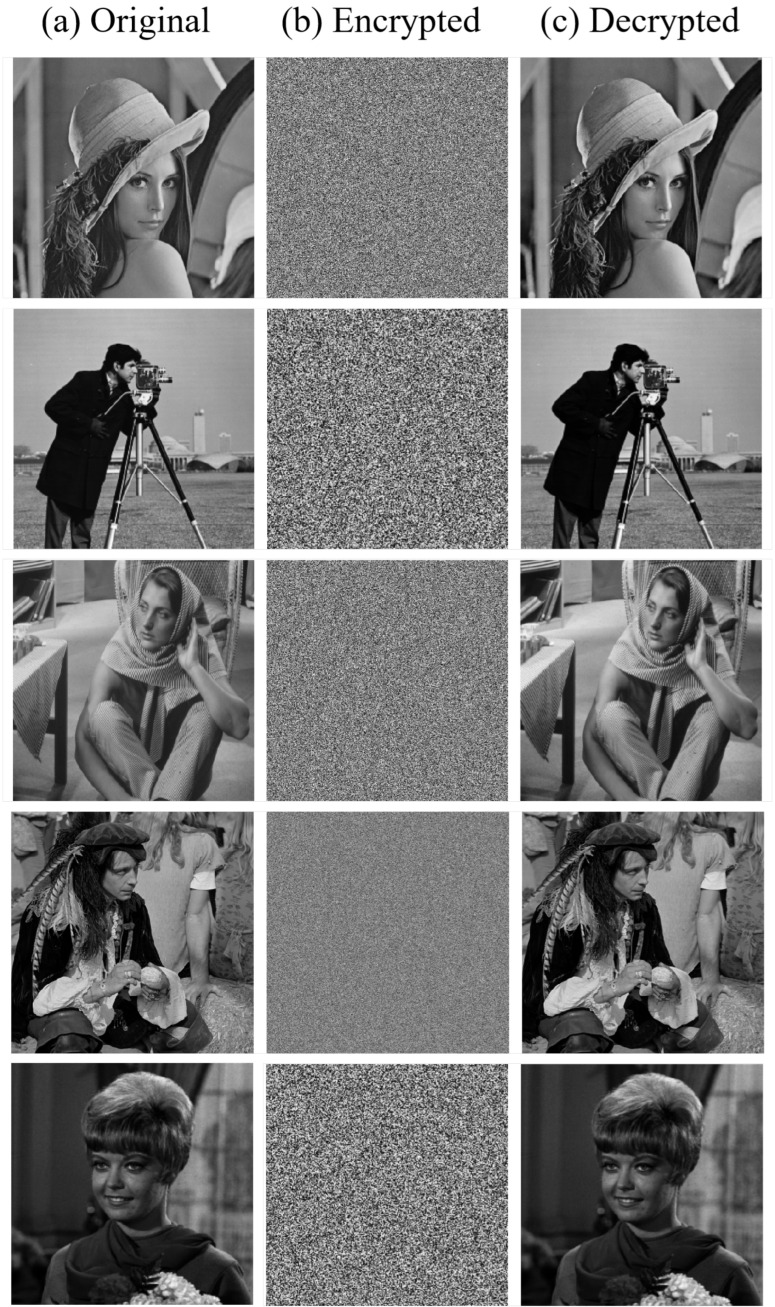
Encryption and decryption results: (**a**) Original; (**b**) Encrypted; (**c**) Decrypted.

Decrypting the facial region involves the key operations below: First, bit order reversal is conducted: using the same chaotic sequence employed during encryption, a bit reversal matrix is generated. An XOR operation between this matrix and the encrypted facial image is then performed to recover the original bit sequence. Second, the diffusion process is reversed: the chaotic matrix used in the encryption phase is regenerated, and an XOR operation with this matrix is executed to restore the facial region to its pre-diffusion state. To fully restore the facial region image, the bit-reversed image must undergo a third core operation: inverse DNA encoding. This involves applying the corresponding decoding method based on the rule index used during encryption. Subsequently, an inverse scrambling process is performed on the decoded image, ultimately yielding the decrypted facial image. Ultimately, the decrypted facial and non facial region images are integrated to obtain the complete decrypted image. The final encryption and decryption effects of different images are shown in Figure 11.

## 5. Experimental Results

This section conducts a performance analysis of the proposed encryption model, covering both security and efficiency. The computations were performed on a computer equipped with an Intel(R) Core(TM) Ultra 7 255H (2.00 GHz) processor, 32.0 GB of memory, and Windows 11. The environment was built using Python 3.10.11, utilizing simulation libraries such as time, random, math, hashlib, numpy 2.1.3, matplotlib 3.10.7, opencv-python 4.11.0.86, mtcnn 1.0.0, tensorflow 2.19.0 and keras 3.10.0 to establish the simulation environment. The initial parameters for the encryption model were set as x=y=z=0.1, with control parameters α=0.7 and β=0.9.

### 5.1. Key Space Analysis

The key space refers to the set of all possible keys in an encryption algorithm, and its size (i.e., the number of keys) is determined by the key length and the character set used. Undoubtedly, an increase in the key space enhances security, as it exponentially raises the difficulty for attackers to find the correct key through brute-force attacks. In the constructed encryption model, there are two algorithms: the encryption algorithm for portrait regions and that for non-portrait regions. An innovative key format is adopted for the non-portrait region, which consists of an array of three integers. Each integer can take one of 256 possible values, resulting in a key space of $2^{24}$. The facial region employs a more complex dual-key system: a public key and a private key, both represented as 512-bit binary arrays, thus having a key space of $2^{512}$. The combination of the key spaces of these two encryption algorithms forms a more robust overall key space, which is $2^{536}$. This cumulative space exceeds the key spaces of previous algorithms [46,47,48,49] in Table 4, indicating that our encryption model has an extremely large key space and can provide an extremely high level of security.

For high-similarity images, the effective key space is calculated as follows:

The 3D-MNFC system maintains a strong chaotic state only when α∈(0.2,0.8) and β∈(0.5,1.5). The ratio of effective chaotic parameters $R_{chaos}$ is calculated as 0.3. Let *S* denote the image similarity, and let P(S) denote the key collision probability (P(0.85)=0.1). The collision-free ratio is $R_{\text{no-collision}}=1-P\left(S\right)$. The effective key space is

(12) $$K_{\mathrm{eff}}\left(S\right)=K_{\mathrm{theo}}\times R_{\mathrm{chaos}}\times R_{\text{no-collision}}.$$

For example, when S=0.85, $K_{\mathrm{eff}}\left(S\right)=2^{536}\times 0.3\times 0.9\approx 2^{534}$. It is far larger than the brute-force attack threshold ($2^{128}$), confirming the system’s security even for high-similarity images.

### 5.2. Information Entropy Analysis

Information entropy serves as a pivotal metric for evaluating the security of encrypted images. It quantifies the inherent uncertainty and randomness in ciphertext images, thereby effectively gauging the extent to which an encryption algorithm obscures the statistical properties of the original image. For an 8-bit grayscale image (pixel values range from 0 to 255), the maximum theoretical information entropy is 8 bits. When the entropy of the encrypted image approaches 8, there is a highly chaotic distribution of pixels. This is a characteristic that complicates attackers’ efforts to deduce details of the original image through statistical analysis. The calculation method is as follows (13):

(13) $$H\left(x\right)=-\sum_{i=1}^{N}p_{i}\times log_{2}p_{i},$$

where $p_{i}$ is the frequency of occurrence of each pixel value (0–255) in the ciphertext image. Through calculations, the information entropy results of various tested images are summarized in Table 5. Furthermore, Table 6 compares the entropy of the proposed encryption scheme and other schemes [50,51,52,53,54] after encrypting the Lena image. Importantly, our proposed model achieves the highest information entropy among those schemes. The finding demonstrates its ability to stand up to more advanced network attacks.

### 5.3. Encryption Efficiency Analysis

The proposed model is designed to enhance security and effectively reduce encryption time. As a result, we calculate its working time and compare it with other encryption schemes [33,55,56,57,58] in Table 7. Additionally, the scheme originally applied for facial regions is also compared with the dual-region model. In the comparison, the time costs of both the dual-region encryption and the full-image encryption include the loading and initialization time of MTCNN and VGG16, thus avoiding comparison bias caused by model startup overhead. After encrypting various initial images with both methods, we found that the proposed model takes less time to complete the process. The detailed data are shown in Table 8. These results fully illustrate that the dual-region model achieves significant improvements in encryption efficiency, making it balance data security with fast processing capabilities.

### 5.4. Correlation Analysis

The level of association between pixels in an image serves as a pivotal metric in assessing the robustness of an encryption algorithm. In natural images, adjacent pixels typically demonstrate a strong correlation in horizontal, vertical, and diagonal orientations. This link is often embraced by a correlation coefficient close to 1. A successful encryption scheme aims to disrupt this correlation by approaching a correlation coefficient of 0. To obtain a full picture of how pixels depend on each other in an image’s spatial domain, we should calculate the covariance and standard deviation of the pixel sequence. This lets us derive the correlation coefficient and analyze those three directions we mentioned earlier. The two formulas are given, respectively, as follows (14) and (15):

(14) $$Cov\left(x,y\right)=\frac{1}{N}\sum_{i=1}^{N}\left(x_{i}-\bar{x}\right)\left(y_{i}-\bar{y}\right),$$

(15) $$r_{xy}=\frac{Cov(x,y)}{\sqrt{D(x)\times D(y)}},$$

where *x*, *y* represent two random variables, $x_{i}$ and $y_{i}$ are the i-th sample observation values of them, $\bar{x}$ and $\bar{y}$ are the sample means of *x* and *y*, *N* is the number of samples, and D(x) and D(y) are the sample variances respectively.

**Figure 12:**
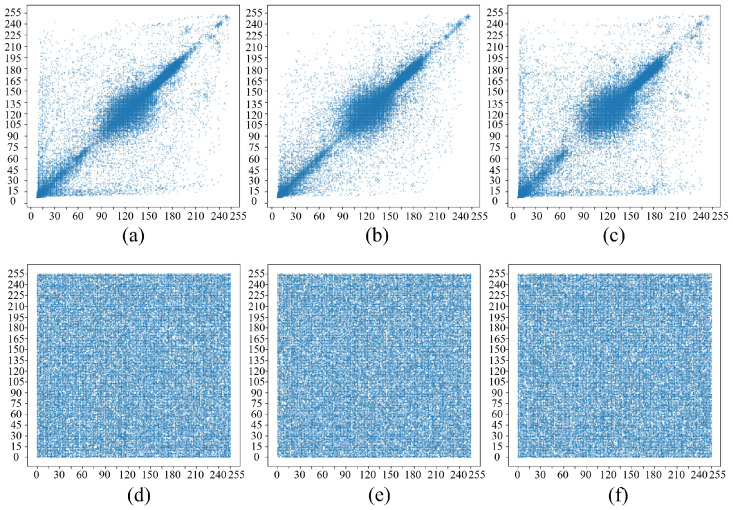
Distribution of the correlation between adjacent pixels in different directions: (**a**–**c**) Original image (horizontal, vertical, diagonal); (**d**–**f**) Encrypted image (horizontal, vertical, diagonal).

If the correlations in the horizontal, vertical, and diagonal directions are significantly reduced after encryption, it indicates that the encryption algorithm effectively destroys the original pixel correlation, greatly increasing the difficulty of resisting statistical attacks. Figure 12 shows the correlation distribution of two adjacent pixels for three directions of Cameraman, and the specific data are recorded in Table 9. The results vividly illustrate the model’s outstanding performance in dismantling statistical pixel correlations.

### 5.5. Histogram Analysis

With the aim of assessing the pixel distribution characteristics of natural images, uniformity analysis places particular focus on the tendency of grayscale values to cluster in specific regions, such as the concentration of skin-tone grayscale values in portrait images. This method generates histograms for the original and encrypted images, then uses these histograms to count how many pixels of each grayscale level. The uniformity of this distribution is quantified by the histogram uniformity coefficient. The ideal value should approach 1/256. A high uniformity indicates that encryption has effectively disrupted the pixel distribution and has hidden statistical features.

**Figure 13:**
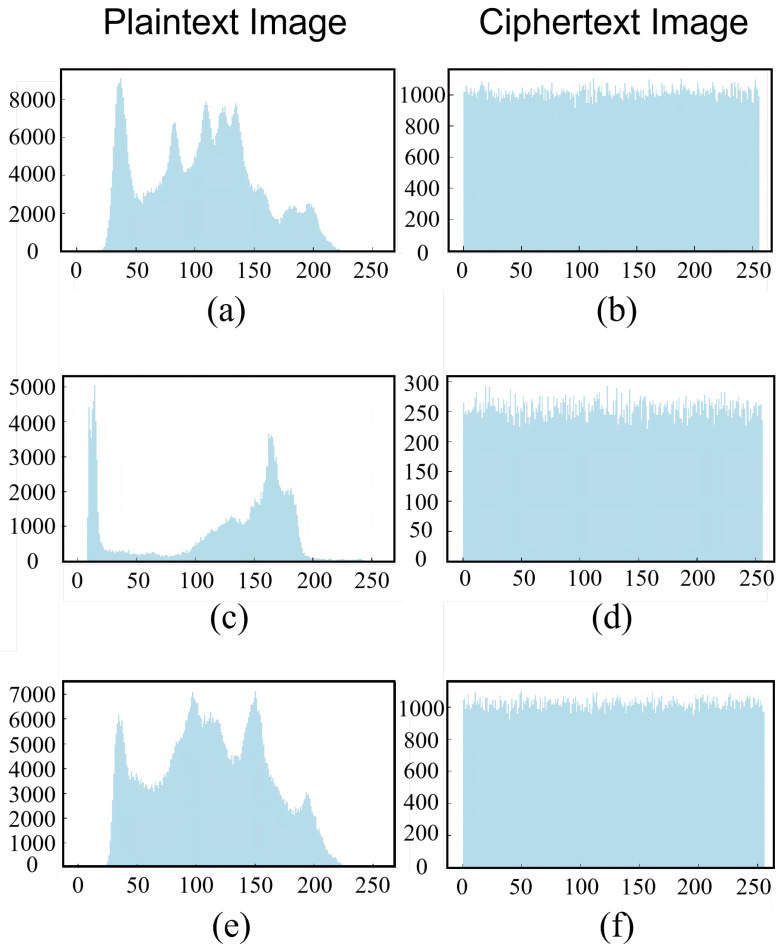
Histograms of original and encrypted images: (**a**) Lena (original); (**b**) Lena (encrypted); (**c**) Cameraman (original); (**d**) Cameraman (encrypted); (**e**) Barbara (original); (**f**) Barbara (encrypted).

Figure 13 is the histograms of different images encrypted by our proposed encryption model. This outcome highlights the model’s effectiveness in breaking down the pixel clustering patterns inherent in the original image and significantly enhancing the statistical randomness of the encrypted image. This strengthens its resistance to statistical analysis attacks successfully.

### 5.6. Avalanche Effect Analysis

The avalanche effect refers to a phenomenon where a tiny change in the initial conditions leads to a drastic alteration in the encryption output. Standards like Number of Pixel Change Rate (NPCR) and Unified Average Changing Intensity (UACI) are used to reflect it. They, respectively, employ formulas detailed in (16) and (17):

(16) $$NPCR\left(img1,img2\right)=\frac{1}{MN}\sum_{i=1}^{M}\sum_{j=1}^{N}diff_{ij}\times 100\%,$$

(17) $$UACI\left(img1,img2\right)=\frac{1}{MN}\sum_{i=1}^{M}\sum_{j=1}^{N}\frac{\left|img1(i,j)-img2(i,j)\right|}{255}\times 100\%,$$

where *M* and *N* are the height and width of the original image. The encrypted image (img1) undergoes a single bit flip to obtain a new encrypted image (img2). $diff_{ij}$ is the pixel difference at position (*i*, *j*) between img1 and img2.

In this encryption model, the encryption algorithm for the portrait region, as the core content, must achieve indicators infinitely close to the ideal ones. For a 256 × 256 image, at a certain significance level, the values of NPCR and UACI are expected to be 99.6094% and 33.4635%. After randomly modifying one bit of the Lena plaintext image and calculating these two indicators, our results show that the NPCR value reaches 99.6055% and the UACI value is 33.4599% for Lena. And the values of other images are illustrated in Table 10. These findings underscore the encryption scheme’s remarkable differential sensitivity and robust diffusion capabilities. Moreover, we also performed calculations on the non-facial regions. For the non-facial region of the Lena image, the NPCR value reaches 99.5775% and the UACI value stands at 31.7058%. Although these values are not as close to the ideal benchmarks as those of the facial region, they are more than sufficient to meet the encryption requirements for this non-sensitive part of the image.

### 5.7. Robustness Analysis

Robustness refers to the ability of an algorithm to maintain its encryption function and security in the face of various attacks, interferences, or abnormal situations, and it is directly related to the effectiveness of data encryption. Resistance to cropping attacks and resistance to noise attacks are important components of it, as these two capabilities mainly reflect the algorithm’s anti-interference performance when facing specific cropping attacks and noise interferences. Peak Signal-to-Noise Ratio (PSNR) provides an objective standard for measuring the ability to resist cropping and noise attacks. It is calculated based on Mean Squared Error (MSE), with Formulas (18) and (19) respectively:

(18) $$MSE=\frac{1}{MN}\sum_{x=0}^{M-1}\sum_{y=0}^{N-1}{\left[I\left(x,y\right)-K\left(x,y\right)\right]}^{2},$$

(19) $$PSNR=10log_{10}\left(\frac{MAX_{I}^{2}}{MSE}\right).$$

The pixel values at coordinates (*x*, *y*) in the original image and the cropped version are denoted as I(x,y) and K(x,y), respectively. The dimensions of the image are given by its height *M* and width *N*. The highest pixel value in the original image is noted as $MAX_{I}$.

**Figure 14:**
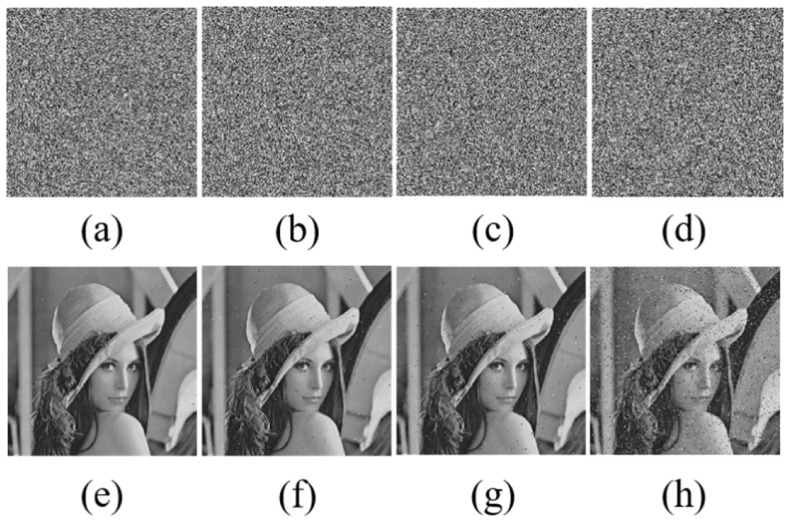
Ciphertext images under noise attacks with densities of (**a**) 0.001, (**b**) 0.005, (**c**) 0.01, and (**d**) 0.1 and their corresponding decrypted images (**e**–**h**).

**Figure 15:**
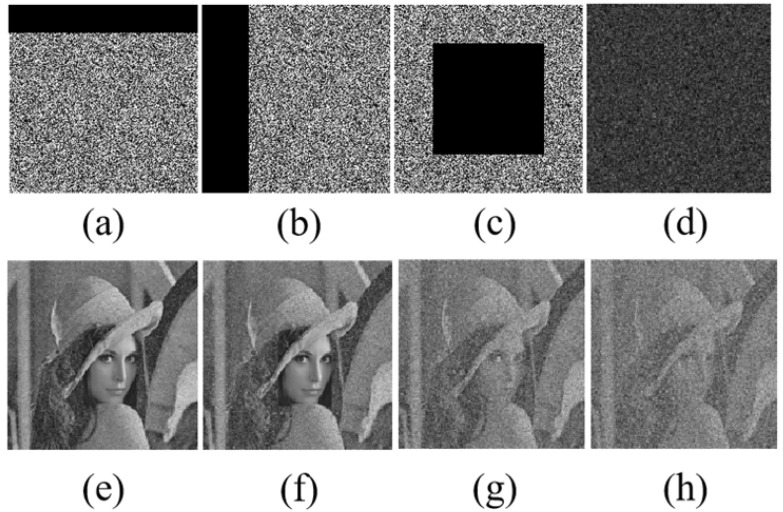
Ciphertext images under cropping attacks with ratios of (**a**) 15%, (**b**) 25%, (**c**) 35%, and (**d**) 55% and their corresponding decrypted images (**e**–**h**).

The image was subjected to cropping attacks with different size ratios and noise attacks with different densities. Figure 14 and Figure 15 further show the restoration effect of the Lena image after noise and cropping attacks. According to the results, the image can effectively resist various interferences. Therefore, this algorithm is suitable for scenarios that need to resist multiple attacks and can effectively ensure the recovery effect of images after being interfered with during transmission in practical applications.

### 5.8. Results in Real Scenarios

**Figure 16:**
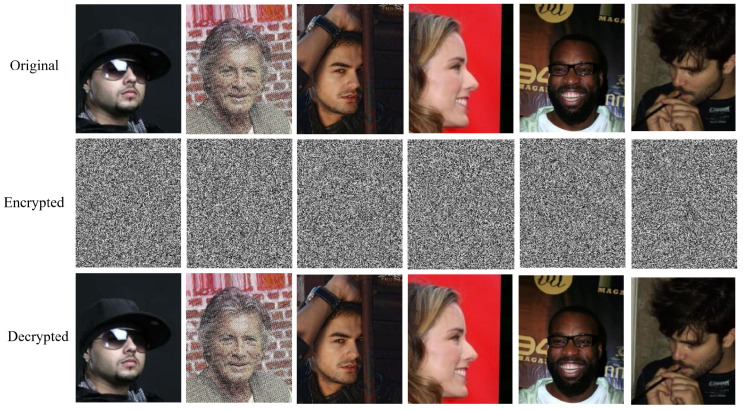
Results of different real-scene facial images.

To verify the usability of the proposed model, we selected some low-resolution images with occlusions and other common artifacts that may appear in real scenarios from the CelebA dataset for testing. The test results are shown in Figure 16, where all the selected images were successfully encrypted and decrypted. This indicates that the proposed model can be applied well in practical scenarios.

## 6. Conclusions

This study proposes a dual-region encryption model specifically designed for portrait images. It leverages MTCNN to detect human faces in images and store their positional data. The data are then used to divide the image into two regions: the facial region and the non-facial region. To correlate the key with image features, we adopt a CNN-based key generator to produce a public key, which is further utilized to create the master key. Given the inflexible parameters of existing chaotic systems, we put forward a novel chaotic system named the 3D-MNFC. We theoretically analyze the periodicity avoidance capability of the 3D-MNFC system, which enhances the unpredictability of the chaotic system. Meanwhile, tests demonstrate that the 3D-MNFC system achieves favorable Lyapunov exponents and permutation entropy and passes the NIST randomness test, all of which verify its excellent dynamic characteristics.

For the facial region, we employ an encryption scheme combining the 3D-MNFC with scrambling, DNA encoding, diffusion, and bit inversion. This multi-layered process fully ensures the security of the facial sensitive region. The encryption of the non-facial region relies on the logistic map and XOR operation, and this lightweight encryption is intended to improve the overall encryption efficiency. After encryption, the two regions are restored to their original positions using the stored positional information. Decryption is performed in the reverse order. We conduct an analysis of the model’s security and efficiency. Evaluation results show that the model exhibits strong resistance against various attacks and can securely encrypt portrait images. We also calculate the efficiency of the proposed model. The results confirm that the dual-region method is more efficient and demonstrate that the model can effectively balance security and efficiency.

In most scenarios, identity-representative information is contained in the facial region, so this study mainly focuses on the design and analysis of the facial region. For cases where information such as clothing in the non-facial region can also indicate identity, the encryption model proposed in this paper has certain limitations, and we will create a more comprehensive algorithm design in the future. In addition, the idea of region-based encryption proposed in this paper can be extended to other general types of images, which requires more precise segmentation techniques to divide the core regions for achieving secure and efficient encryption.

## Figures and Tables

**Table 1:** DNA encoding rules.

Base	Rule 1	Rule 2	Rule 3	Rule 4	Rule 5	Rule 6	Rule 7	Rule 8
A	00	00	01	01	10	10	00	00
T	11	11	10	10	01	01	11	11
C	01	10	00	01	00	10	01	10
G	10	01	11	11	10	00	10	01

**Table 2:** The results of NIST tests.

Test	X-seq	Y-seq	Z-seq	Result
Approximate Entropy	0.536292	0.493724	0.299761	Success
Block Frequency	0.269568	0.373244	0.154652	Success
Cumulative Sums	0.062363	0.127765	0.019553	Success
Discrete Fourier Transform Test	0.425317	0.3660778	0.193279	Success
Frequency	0.161927	0.462949	0.325863	Success
Linear Complexity	0.617653	0.882476	0.415933	Success
Longest Run	0.497203	0.353481	0.337534	Success
Non Overlapping Template	0.517501	0.565158	0.494387	Success
Overlapping Template	0.885625	0.901368	0.804579	Success
Runs Test	0.422496	0.795367	0.544602	Success
Random Excursions	0.525860	0.672229	0.372714	Success
Random Excursions Variant	0.729659	0.490996	0.088815	Success
Rank	0.362564	0.093562	0.026571	Success
Serial	0.863365	0.712044	0.052736	Success
Universal	0.188326	0.155473	0.027359	Success

**Table 3:** Robustness test results of face detection.

Image	Original Image	Low Brightness	High Brightness	Low Contrast	High Contrast	Gaussian Noise
Lena	1	1	1	1	1	1
Barbara	1	0	1	1	1	1
Cameraman	1	1	1	0	1	1
Female	1	1	1	1	1	1
Male	1	0	1	1	1	1

**Table 4:** Key space of different schemes.

Scheme	Proposed	[46]	[47]	[48]	[49]
Key space	$2^{536}$	$2^{256}$	$2^{272}$	$2^{419}$	$2^{469}$

**Table 5:** Information entropy of the images through the proposed encryption model.

Image	Plaintext Image	Ciphertext Image
Lena	7.4331	7.9995
Barbara	7.5662	7.9995
Cameraman	7.0097	7.9992
Female	7.0526	7.9996
Male	7.5237	7.9998

**Table 6:** Comparison of information entropy of the encrypted Lena image.

Scheme	Proposed	[50]	[51]	[52]	[53]	[54]
Entropy	7.9995	7.9992	7.9991	7.9987	7.9993	7.9994

**Table 7:** A comparison of time efficiency between the proposed model and other schemes.

Scheme	Proposed	[55]	[33]	[56]	[57]	[58]
Time(s)	1.07	4.27	3.06	1.42	1.24	1.12

**Table 8:** Improvement in the time efficiency of the encryption model.

Image	Dual-Region Encryption	Full-Image Encryption	Improvement
Lena	1.08 s	2.04 s	47.06%
Cameraman	0.93 s	1.5 s	38.00%
Barbara	1.13 s	2.02 s	44.06%
Female	0.97 s	1.56 s	37.82%
Male	1.37 s	3.63 s	62.26%

**Table 9:** Correlation statistics before and after encryption.

Direction	Lena		Cameraman		Barbara	
	Original Image	Encrypted Image	Original Image	Encrypted Image	Original Image	Encrypted Image
Horizontal	0.9747	−0.0008	0.9335	0.0078	0.8597	0.0022
Vertical	0.9863	−0.0008	0.9592	0.0045	0.9591	0.0008
Diagonal	0.9627	0.0016	0.9087	−0.0021	0.8418	−0.0027

**Table 10:** The results of avalanche effect analysis.

Indicator	Lena	Barbara	Cameraman	Female	Male
NPCR	99.6055%	99.6015%	99.5972%	99.6038%	99.5960%
UACI	33.4599%	33.4368%	33.4577%	32.4563%	33.4370%

## Data Availability

The study’s supporting data can be found in USC-SIPI (http://sipi.usc.edu/database (accessed on 22 July 2025)) and CelebA (https://mmlab.ie.cuhk.edu.hk/projects/CelebA.html (accessed on 9 January 2026)).

## Abbreviations

The following abbreviations are used in this manuscript:

3D-MNFC	Three-dimensional Multi-module Nonlinear Feedback-coupled Chaotic System
CNN	Convolutional Neural Network
MTCNN	Multi-task Cascaded Convolutional Network
NPCR	Number of Pixel Change Rate
UACI	Unified Average Changing Intensity
PSNR	Peak Signal-to-Noise Ratio
MSE	Mean Squared Error
NMS	Non-Maximum Suppression
DES	Data Encryption Standard
AES	Advanced Encryption Standard
PE	Permutation Entropy
